# RIG-I Detects Kaposi’s Sarcoma-Associated Herpesvirus Transcripts in a RNA Polymerase III-Independent Manner

**DOI:** 10.1128/mBio.00823-18

**Published:** 2018-07-03

**Authors:** Yugen Zhang, Dirk P. Dittmer, Piotr A. Mieczkowski, Kurtis M. Host, William G. Fusco, Joseph A. Duncan, Blossom Damania

**Affiliations:** a Lineberger Comprehensive Cancer Center, University of North Carolina at Chapel Hill, Chapel Hill, North Carolina, USA; b Department of Microbiology and Immunology, University of North Carolina at Chapel Hill, Chapel Hill, North Carolina, USA; c High Throughput Sequencing Facility, University of North Carolina at Chapel Hill, Chapel Hill, North Carolina, USA; d Department of Genetics, University of North Carolina at Chapel Hill, Chapel Hill, North Carolina, USA; e Department of Medicine, University of North Carolina at Chapel Hill, Chapel Hill, North Carolina, USA; University of Michigan—Ann Arbor

**Keywords:** Kaposi's sarcoma-associated herpesvirus, RIG-I, innate immunity

## Abstract

Retinoic acid-inducible gene I (RIG-I) is a cytosolic pathogen recognition receptor that initiates the innate immune response against many RNA viruses. We previously showed that RIG-I restricts Kaposi's sarcoma-associated herpesvirus (KSHV) reactivation (J. A. West et al., J Virol 88:5778–5787, 2014, https://doi.org/10.1128/JVI.03226-13). In this study, we report that KSHV stimulates the RIG-I signaling pathway in a RNA polymerase (Pol) III-independent manner and subsequently induces type I interferon (IFN) responses. Knockdown or inhibition of RNA Pol III had no effect on beta interferon (IFN-β) induction by KSHV. By using high-throughput sequencing of RNA isolated by cross-linking immunoprecipitation (HITS-CLIP) approach, we identified multiple KSHV regions that give rise to RNA fragments binding to RIG-I, such as ORF8_10420-10496_, Repeat region (LIR1)_119059-119204_, and ORF25_43561-43650_. The sequence dissimilarity between these fragments suggests that RIG-I detects a particular structure rather than a specific sequence motif. Synthesized ORF8_10420-10496_ RNA stimulated RIG-I-dependent but RNA Pol III-independent IFN-β signaling. In summary, several KSHV RNAs are sensed by RIG-I in a RNA Pol III-independent manner.

## IMPORTANCE

Kaposi’s sarcoma-associated herpesvirus (KSHV) is the causative agent of Kaposi’s sarcoma, primary effusion lymphoma, and multicentric Castleman’s disease. Innate immune responses against viral infections, especially the induction of type I interferon, are critical for limiting the replication of viruses. Retinoic acid-inducible gene I (RIG-I), a cytosolic RNA helicase sensor, plays a significant role in the induction of type I interferon responses following viral infection. Here, we identified multiple RNA regions in KSHV as potential virus ligands that bind to RIG-I and stimulate RIG-I-dependent but RNA Pol III-independent IFN-β signaling. Our results expand the role of RIG-I by providing an example of a DNA virus activating a canonical RNA-sensing pathway.

## INTRODUCTION

The inflammatory response triggered by the cytosolic sensors known as RIG-I-like receptors (RLRs) is one of the first and most important lines of defense against infection. There are three members of the RLR family: retinoic acid-inducible gene I (RIG-I), melanoma differentiation gene 5 (MDA5), and laboratory of genetics and physiology 2 (LGP2). During RNA virus infection, RIG-I recognizes viral RNA fragments, and RNA binding induces conformational changes and oligomerization of RIG-I (1). Activated RIG-I then binds an adaptor protein, mitochondrial antiviral signaling protein (MAVS), on the mitochondrial and peroxisomal membranes, activating multiple downstream signaling molecules, most notably TANK-binding kinase 1 (TBK1) and the IκB kinase (IKK) family of proteins. These, in turn, signal through the transcription factors NF-κB, interferon regulatory factor 3 (IRF3), and IRF7 to produce inflammatory cytokines, including beta interferon (IFN-β) (2–4).

RIG-I signaling is essential for the control of infection by many RNA viruses, such as hepatitis C virus (HCV) (5), vesicular stomatitis virus (VSV) (6), and Japanese encephalitis virus (7). RIG-I preferentially binds to RNA ligands that are short fragments (<300 nucleotides) (6, 8–10). RIG-I also detects RNA fragments that contain 5′ triphosphate (5′ ppp) ends, as well as a RNA panhandle or bulge/loop structures (1, 11–13), suggesting a certain promiscuity with regard to substrate specificity. Recently, immunoprecipitation of RIG-I/virus RNA complexes coupled to next-generation sequencing have allowed for the identification of physiological RIG-I agonists from cells infected with RNA viruses, such as West Nile virus (WNV) (14), influenza A virus (IAV), or Sendai virus (15, 16). Other reports have identified physiologically relevant MDA5 agonists in picornavirus-infected cells (17, 18) and in measles virus (MeV)-infected cells (19), thus validating this experimental approach.

RIG-I has previously been shown to be involved in sensing DNA viruses in a RNA polymerase (Pol) III-dependent manner. RNA polymerase III transcribes cytosolic AT-rich DNA from DNA viruses into RNA that can then serve as a pathogen-associated molecular pattern (PAMP) for RIG-I-mediated immune responses (20, 21). For example, adenovirus induces IFN-β expression in a RNA polymerase III-dependent manner through activation of RIG-I (22). The herpesvirus Epstein-Barr virus (EBV) (also called human herpesvirus 4 [HHV4])-encoded RNAs (EBERs) are transcribed by RNA polymerase III, and they can activate RIG-I and type I interferons (21, 23). In contrast, sensing of the herpesvirus, herpes simplex virus 1 (HSV-1/HHV1), seems to occur by RNA polymerase III-independent mechanisms (20, 24, 25), suggesting that among the large DNA viruses, multiple pathways can give rise to RIG-I substrates. We wanted to determine whether RIG-I could sense Kaposi’s sarcoma-associated herpesvirus (KSHV/HHV8) and whether this was by a Pol III-dependent or -independent mechanism.

KSHV is a large double-stranded DNA virus that is the etiologic agent of Kaposi’s sarcoma (KS), primary effusion lymphoma (PEL), and multicentric Castleman’s disease (MCD) (26, 27). An increasing body of work indicates that regulation of host immune responses is critical for establishing KSHV infection (28–32). We have previously shown that depletion of either MAVS or RIG-I increased transcription of the viral genome upon *de novo* infection, while beta interferon (IFN-β) induction was attenuated, suggesting that RIG-I was indeed activated and had an inhibitory role during primary infection and during reactivation from latency (33). The mechanism by which RIG-I senses KSHV and the virus-derived ligands that bind to RIG-I following infection were unclear. To address this gap in our knowledge, we used a high-throughput sequencing of RNA isolated by cross-linking immunoprecipitation (HITS-CLIP) approach to identify RNA fragments within the KSHV genome that bind to RIG-I and activate the RIG-I pathway. We also established that RNA polymerase III was not required for KSHV-mediated IFN-β induction.

## RESULTS

### RNA Pol III is not required for IFN-β induction by KSHV.

**FIG 1:**
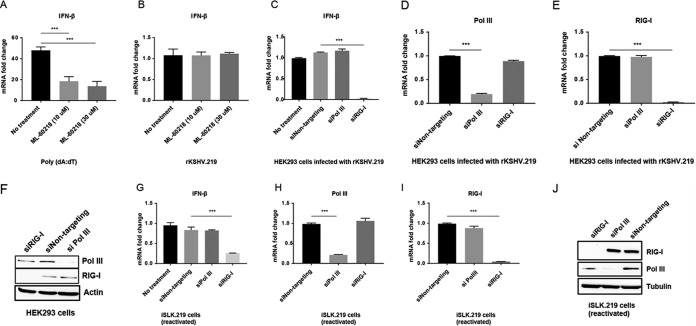
RNA Pol III is not required for IFN-β expression during KSHV primary infection or reactivation. (A and B) HEK293 cells in 24-well plates were treated with the indicated concentrations of the RNA Pol III inhibitor ML-60218. Eight hours after treatment, cells were transfected with poly(dA·dT) for 6 h (A) or infection with rKSHV.219 for 48 h (B), IFN-β mRNA level was measured by qRT-PCR. (C to F) HEK293 cells in 24-well plates were transfected with siRNAs targeting RNA Pol III or RIG-I. Twenty-four hours posttransfection, cells were infected with rKSHV.219 for 48 h. The relative expression levels of IFN-β (C), RNA Pol III (D), and RIG-I (E) normalized to β-actin were measured by qRT-PCR. (F) Western blotting showing efficient knockdown of RNA Pol III or RIG-I in HEK293 cells. (G to J) iSLK.219 cells latently infected with rKSHV.219 were transfected with nontargeting control, RNA Pol III, or RIG-I siRNA. Twenty-four hours after siRNA transfection, doxycycline was added to reactivate the iSLK.219 cells. At 72 h postreactivation, the relative expression levels of IFN-β (G), RNA Pol III (H), and RIG-I (I) normalized to β-actin were measured by qRT-PCR. (J) Western blots showing efficient knockdown of RNA Pol III or RIG-I in iSLK.219 cells. Data are presented as means plus standard deviations (SD). Error bars represent the variation range of duplicate experiments. The data are representative of three independent experiments. Values that are statistically significantly different are indicated by bars and asterisks as follows: **, *P* < 0.01; ***, *P* < 0.001.

RNA polymerase (Pol) III is an intracellular DNA sensor that has been shown to transcribe viral DNA into a RNA intermediate that stimulates RIG-I (21). To determine the role of RNA Pol III in the KSHV-mediated RIG-I pathway, two different approaches were used: Pol III inhibition by a small molecule and Pol III depletion by small interfering RNA (siRNA) transfection. First, we treated HEK293 cells with a specific chemical inhibitor of RNA Pol III, ML-60218, at 10 and 30 µM (34) before transfection with poly(dA-dT) or infection with rKSHV.219 (35). rKSHV.219 is a recombinant virus strain of KSHV that carries red and green fluorescence marker genes (35). As expected, both doses of inhibitor ML-60218 inhibited IFN-β induction in cells transfected with poly(dA-dT) (Fig. 1A). In contrast, IFN-β induction was not affected by the Pol III inhibitor at either dose in cells infected with rKSHV.219 (Fig. 1B). Second, siRNAs targeting RIG-I or RNA Pol III (POLR3A) were transfected into HEK293 cells to deplete the essential RNA Pol III subunit, POLR3A. Cells were then infected with rKSHV.219, and IFN-β was measured at 48 h postinfection. RIG-I siRNA, but not the control nontargeting siRNA, inhibited IFN-β induction (Fig. 1C). In contrast, IFN-β mRNA was not affected by the knockdown of POLR3A in KSHV-infected HEK293 cells (Fig. 1C). The siRNA-mediated knockdowns of Pol III and RIG-I were confirmed by both mRNA and protein expression using quantitative reverse transcription-PCR (qRT-PCR) and Western blotting, respectively (Fig. 1D, E, and F). Next, the role of RNA Pol III was probed by using iSLK.219 cells transfected with siRNA targeting Pol III (POLR3A) or RIG-I, followed by the addition of doxycycline for 72 h to reactivate the virus. Similar results as in HEK293 cells were observed for IFN-β in reactivated iSLK.219 cells (Fig. 1G). Both qRT-PCR and Western blotting confirmed the siRNA-mediated knockdowns of Pol III and RIG-I at both mRNA and protein levels (Fig. 1H, I, and J). Cotransfection with two siRNAs that target two different Pol III subunits, POLR3A and POLR3D, followed by KSHV infection likewise was unable to block IFN-β induction (data not shown). Overall, these data demonstrate that RNA Pol III is not required for IFN-β induction during either primary KSHV infection or reactivation.

### Isolation of RIG-I/RNA complexes from latent and lytic iSLK cells.

**FIG 2:**
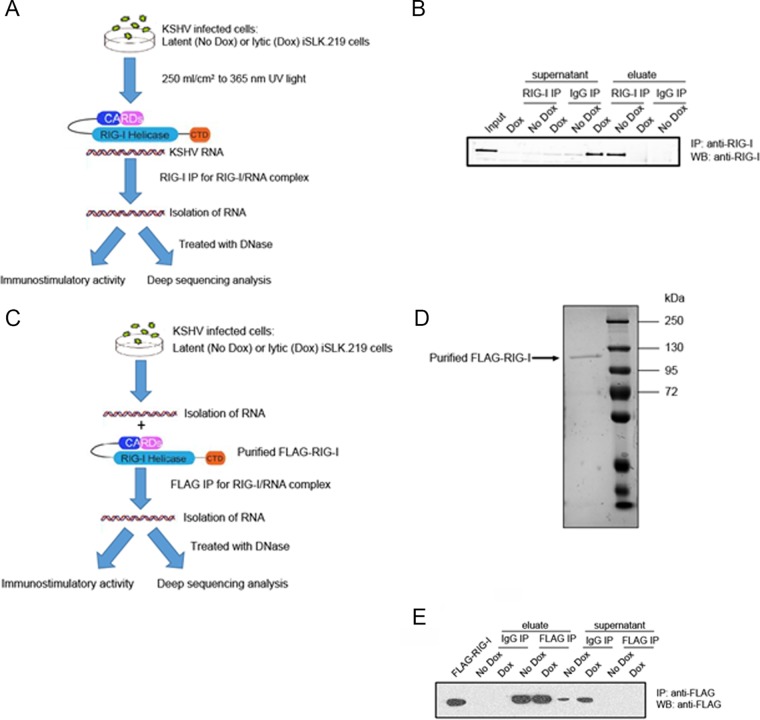
Isolation and characterization of RIG-I/RNA complexes from latent and lytic iSLK cells. (A) Schematic representation of CLIP (cross-linking and endogenous RIG-I immunoprecipitation [IP]) procedure. CARDs, caspase activation and recruitment domains; CTD, C-terminal domain. (B) Western blot (WB) analysis from RIG-I pulldowns shows the efﬁciency and specificity of endogenous RIG-I IP. (C) Schematic representation of purified FLAG-RIG IP procedure. (D) FLAG-tagged RIG-I (FLAG-RIG-I) protein in an 8% polyacrylamide gel stained by an ultrasensitive Coomassie blue stain. (E) Western blot analysis from FLAG pulldowns shows the efﬁciency and specificity of purified FLAG-RIG IP. The Western blots are representative of three independent CLIPs.

To test the hypothesis that viral RNAs directly interact with RIG-I in KSHV-infected cells, we selected the iSLK.219 cell line (36) because all cells are latently infected with KSHV and can be 100% induced by doxycycline, avoiding the need for pleiotropic inducing agents such as phorbol esters and sodium butyrate, which may impact cellular gene expression and broadly affect cell physiology. Fluorescence images of latent (green fluorescent protein [GFP]) and reactivated (red fluorescent protein [RFP]) iSLK.219 cells show that almost every cell (>95%) was RFP positive after doxycycline treatment (see Fig. S1 in the supplemental material). Although it is well established that RIG-I binds RNA fragments, the affinity between RIG-I and its ligand is low, which may result in very few RNA molecules that copurify with RIG-I during immunoprecipitation. To increase the efficiency of the purification procedure, we applied a cross-linking and immunoprecipitation (CLIP) approach based on photoactivatable-ribonucleoside-enhanced cross-linking (37). Here, live cells were exposed to UV light at 250 mJ/cm in a cross-linker before lysis. Figure 2 outlines the overall schematic for the two experimental procedures: (i) precipitation of endogenous RIG-I and (ii) precipitation of Escherichia coli-produced recombinant RIG-I incubated *in vitro* with iSLK.219 total RNA. Endogenous RIG-I/RNA complexes were immunoprecipitated with a monoclonal antibody against RIG-I. For a control, we used an anti-IgG antibody in parallel throughout the immunoprecipitation (IP) procedure (Fig. 2A). As seen in Fig. 2B, RIG-I was absent in the supernatant (clear solution above the pellet of magnetic beads) and present in the elution from anti-RIG-I magnetic beads (eluate) but not from the control IgG precipitation. Figure 2C outlines the overall schematic of the purified RIG-I IP procedure in which RNA extracted from latent or lytic iSLK.219 cells was incubated *in vitro* with E. coli-purified FLAG-tagged RIG-I, and RIG-I-associated RNA was immunoprecipitated with anti-FLAG monoclonal antibody. Figure 2D shows the purity of the FLAG-tagged RIG-I protein in a polyacrylamide gel stained with an ultrasensitive Coomassie blue stain. The Western blot shown in Fig. 2E confirmed the efficiency and specificity of this IP.

10.1128/mBio.00823-18.1 FIG S1  Fluorescence images of latent (GFP) and reactivated (RFP) iSLK.219 cells. Virus reactivation in iSLK.219 cells was induced for 48 h by 0.2 µg/ml doxycycline treatment, and the extent of reactivation was measured by detection of RFP-positive cells using high-content fluorescence imaging Download FIG S1, TIF file, 1.5 MB. Copyright © 2018 Zhang et al. 2018 Zhang et al. This content is distributed under the terms of the Creative Commons Attribution 4.0 International license.

### Immunostimulatory activity analysis of RIG-I-associated RNA in WT MEF and RIG-I knockout MEF cells.

**FIG 3:**
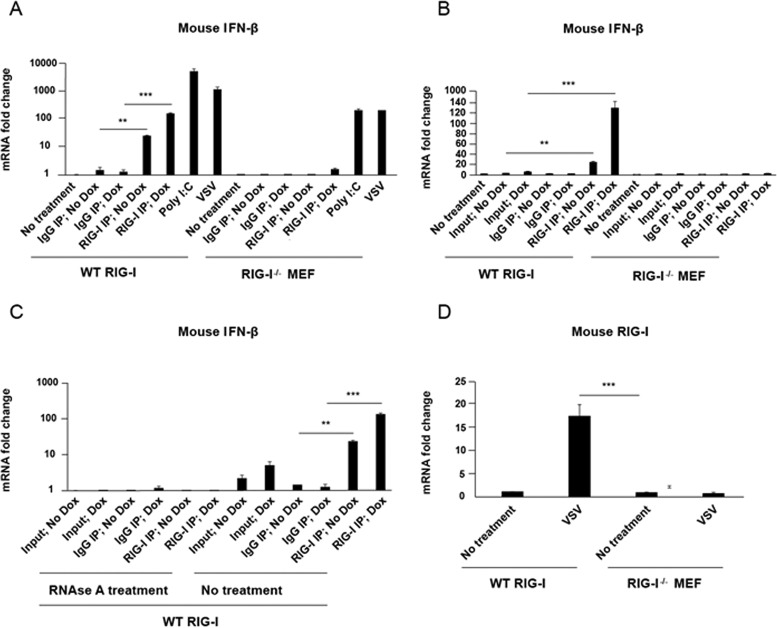
Immunostimulatory activity of RNA from RIG-I and control (IgG) IPs in wild-type (WT) RIG-I and RIG-I knockout MEF cells. WT RIG-I and RIG-I knockout MEF cells in 24-well plates were transfected with four different immunoprecipitated RNA samples: (i) IgG antibody pulldown from latent iSLK.219 cells (IgG IP; No Dox); (ii) IgG antibody pulldown from lytic iSLK.219 cells reactivated with doxycycline (Dox) (IgG IP; Dox); (iii) RIG-I antibody pulldown from latent iSLK.219 cells (RIG-I; No Dox); and (iv) RIG-I antibody pulldown from lytic iSLK.219 cells (RIG-I IP; Dox). In parallel, cells were transfected with poly(I·C) or infected with VSV (positive controls). Twenty-four hours after RNA transfection or virus infection, total RNA was purified from transfected or infected cells and was treated with RNase A or not treated with RNase A, followed by qRT-PCR analysis for mouse IFN-β mRNA. (A) Enrichment of immunostimulatory RNA from RIG-I IP Dox compared with control (IgG) IP. (B) Enrichment of immunostimulatory RNA with RIG-I IP compared with total RNA (Input) from latent iSLK.219 cells (No Dox) or lytic iSLK.219 cells (Dox). (C) RNase A treatment of RIG-I-bound RNA as well as Input RNAs completely abolishes their immunostimulatory activity. (D) Mouse RIG-I mRNA in WT RIG-I and RIG-I^−/−^ MEF by qRT-PCR analysis to confirm that there is no RIG-I in RIG-I^−/−^ MEF. Data are presented as mean ± SD. Error bars represent the variation range of duplicate experiments. The data are representative of three independent experiments. **, *P* < 0.01; ***, *P* < 0.001.

To establish the biological activity of the RIG-I copurifying RNA fragments, the bound RNA was tested for immunostimulatory activity. RNAs derived from the following samples were compared: (i) IgG antibody immunoprecipitated from latent iSLK.219 cells with no doxycycline (Dox) (IgG IP; No Dox); (ii) IgG antibody immunoprecipitated from lytic iSLK.219 cells reactivated with doxycycline (IgG IP; Dox); (iii) RIG-I antibody immunoprecipitated from latent iSLK.219 cells (RIG-I IP; No Dox); and (iv) RIG-I antibody immunoprecipitated from lytic iSLK.219 cells (RIG-I IP; Dox). Purified RNA was transfected into wild-type (WT) and RIG-I knockout (RIG-I^−/−^) mouse embryo fibroblasts (MEF). Poly(I·C) transfection and VSV infection served as positive controls. Twenty-four hours after RNA transfection or virus infection, total RNA was purified, and mouse IFN-β mRNA levels were determined by qRT-PCR. As seen in Fig. 3A, RNA from sample “RIG-I IP; Dox” significantly increased mouse IFN-β mRNA levels compared to RNA from sample “IgG IP; Dox” and RNA from sample “RIG-I IP; No Dox” (note the logarithmic scale). No IFN-β mRNA was induced in response to either sample in RIG-I^−/−^ MEF, demonstrating that the IFN-β induction was dependent on RIG-I. Poly(I⋅C) and VSV induced IFN-β mRNA independent of RIG-I status presumably via TLR3-TRIF (TLR3 is Toll-like receptor 3, and TRIF is Toll/interleukin 1 receptor domain-containing adaptor-inducing beta interferon).

To test the hypothesis that the immunostimulatory activity of RNA from KSHV-reactivated cells was enriched by the RIG-I IP, WT MEF and RIG-I^−/−^ MEF cells were transfected with RNA from the RIG-I immunoprecipitation and total RNAs from latent iSLK.219 cells (Input; No Dox) and reactivated iSLK.219 cells (Input; Dox). There was significant induction of IFN-β in response to RNA from the RIG-I IP but only a moderate amount of IFN-β induction from Input RNA (Fig. 3B). This demonstrates that RIG-I immunoprecipitation enriches the immunogenic activity of the RNA generated from iSLK.219 cells to induce IFN-β activity and that KSHV reactivation increases this activity.

Finally, to confirm that it was RNA, not DNA, that induced IFN-β activity when transfected into WT or RIG-I^−/−^ MEF, the nucleic acids from the IP complexes were treated with RNase A before transfection into cells. As shown in Fig. 3C, RNase A treatment abolished all immunostimulatory activity, confirming that the PAMPs associated with RIG-I in KSHV-infected iSLK.219 cells were indeed RNA molecules. Figure 3D validates the RIG-I knockout, showing that there was no RIG-I mRNA detected in RIG-I^−/−^ MEF even after VSV infection. Taken together, these data show that RIG-I CLIP concentrates IFN-β-inducing RNAs from KSHV-infected cells and these RNAs are upregulated by viral reactivation.

### Deep sequencing reveals RNAs derived from KSHV that bind to RIG-I.

**FIG 4:**
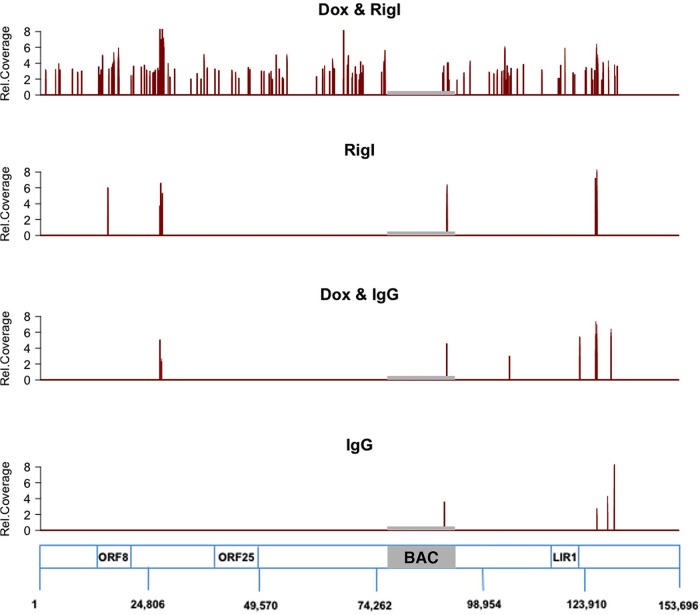
KSHV RNAs are selectively enriched in RIG-I immunoprecipitates compared to control IgG immunoprecipitates by deep sequencing analysis. RNAs from RIG-I pulldown and control (IgG) pulldown from both latent and lytic iSLK.219 cells were subjected to Illumina deep sequencing analysis. Reads were aligned to KSHV JSC-1 isolated clones into BAC16 (GQ994935) and verified by whole-genome sequencing. Relative coverage is shown on the *y* axes. Further details are in Fig. S5 in the supplemental material.

To test the hypothesis that some of the RNAs that bound to and copurified with RIG-I were of viral origin, we carried out Illumina sequencing on the isolated RNA species from RIG-I IP or control IgG IP from uninduced or induced iSLK219 cells. Prior to library preparation, the released RNA was enriched for small RNA species (<300 nucleotides), since RIG-I is known to bind small RNAs. Reads were mapped to the KSHV genome, and relative abundances between RIG-I IP and GFP control IP, as well as total RNA from latent iSLK.219 cells (No Dox) and lytic iSLK.219 cells (Dox), were compared. Figure 4 is a graphical representation of sequences mapped to the JSC-1 isolate of KSHV cloned as a bacmid (GenBank accession no. GQ994935.1). Individual peaks on the graph correspond to a sequencing read that starts at that particular position mapped to the KSHV genome, and the *x* axis corresponds to all possible positions in the KSHV genome. The distribution of sequences in the RIG-I IP; Dox sample displayed a large number of discrete peaks across the KSHV genome, while IgG; Dox IP samples had very few peaks across the KSHV genome (Fig. 4), indicating that the RNA in the RIG-I IP is specifically enriched for KSHV-derived sequences. The IgG IP; No Dox and RIG-I IP; No Dox samples from latent iSLK.219 cells showed very few peaks across the KSHV genome (Fig. 4 and Fig. S5). A comparison to total RNA or small RNA enriched from Dox-treated cells showed that during reactivation in iSLK.219 cells, the entire KSHV genome was transcribed. This suggests that during lytic replication, several KSHV RNA intermediates can be recognized by RIG-I.

The positions of some KSHV sequences identified to bind RIG-I in at least three independent biological replicates are shown in Table 1. Figure S5 shows the results for an additional two biological replicates, where each transcriptome sequencing (RNA-seq) library was split across two Illumina lanes to reduce technical batch effects. Out of these candidate fragments, we focused on several high-confidence KSHV-derived sequences that were immunoprecipitated exclusively by the RIG-I antibody but not by the IgG antibody.

**TABLE 1:** Positions in the KSHV genome of the KSHV fragments identified by Illumina deep sequencing analysis

Fragment name	Location in genome (nucleotide positions^a^)
ORF4	2,162–2,530
ORF8	10,420–10,569
ORF11	16,112–16,241
K2	17,792–17,834
ORF22	37,181–37,373
ORF25	43,561–43,650
ORF46	68,636–68,889
K8	74,774–74,965
Repeat region (LIR1)	119,059–119,204
ORF75	133,617–133,712

a The nucleotide positions are based on the sequence from GenBank accession number GQ994935.1 for the JSC-1 bacterial artificial chromosome (BAC).

### Confirmation of RIG-I-associated KSHV RNA fragments by qRT-PCR.

**FIG 5:**
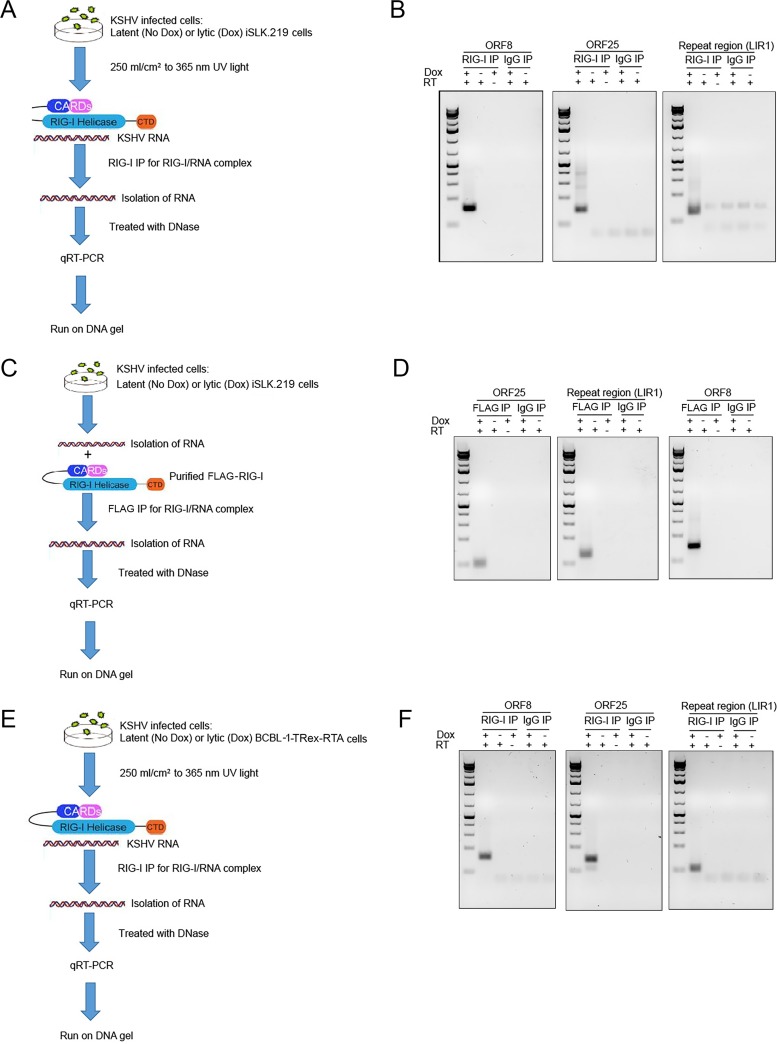
qRT-PCR analysis of RIG-I-associated RNA from RIG-I and control (IgG) IPs. (A) Schematic of qRT-PCR procedure from endogenous RIG-I immunoprecipitation (IP) in latent or lytic iSLK cells. (B) qRT-PCR products of RNAs immunoprecipitated from endogenous RIG-I IP were run on an agarose gel, revealing KSHV-specific amplifications not present in the control IgG IP. The presence (+) or absence (−) of doxycycline (Dox) and reverse transcriptase (RT) are shown above the lanes. (C) Schematic of qRT-PCR procedure from purified recombinant FLAG-RIG-I IP in latent or lytic iSLK cells. (D) qRT-PCR products of RNAs immunoprecipitated from recombinant FLAG-RIG-I were run on an agarose gel, revealing KSHV-specific amplifications not present in the control IgG IP. (E) Schematic of qRT-PCR procedure from endogenous RIG-I IP in latent and lytic BCBL1 cells. (F) qRT-PCR products from RNAs immunoprecipitated from endogenous RIG-I IP of lytic BCBL1 cells were run on an agarose gel, revealing KSHV-specific gene bands not present in the control IgG IP. The data are representative of three independent qRT-PCR experiments.

To validate the deep sequencing results, RT-PCR amplification was performed independently on three of the top 15 KSHV genes identified by RNA deep sequencing analysis. The KSHV segments ORF8_10420-10496_, Repeat region (LIR1)_119059-119204_, and ORF25_43561-43650_ were selected for qRT-PCR assay, because multiple discrete peaks were concentrated in these regions across multiple experiments. The locations of these genes in the KSHV genome are shown in Table 1, though there is some variability (within 100 bp), which we attribute to linker ligation efficiency. The qRT-PCR procedure is illustrated in Fig. 5A. Briefly, total RNA was purified from latent (No Dox) or reactivated (Dox) iSLK.219 cells and subjected to immunoprecipitation with RIG-I antibody or control IgG antibody as described previously. RNA was treated with RNase-free DNase, and then processed for reverse transcription and quantitative PCR (qPCR). The primer sequences are shown in Table S1. When the qRT-PCR products were analyzed, sequences located within KSHV ORF8_10420-10496_, Repeat region (LIR1)_119059-119204_, and ORF25_43561-43650_ were found in the endogenous RIG-IP from lytic iSLK.219 cells, but not from latent iSLK.219 cells or IgG IP samples (Fig. 5B). There was no signal in RIG-I IP lytic cell samples without reverse transcriptase, indicating that signal was due to RNA, not contaminating DNA (Fig. 5B).

10.1128/mBio.00823-18.6 TABLE S1  DNA oligonucleotide primers used in qRT-PCR. Download TABLE S1, DOCX file, 0.02 MB. Copyright © 2018 Zhang et al. 2018 Zhang et al. This content is distributed under the terms of the Creative Commons Attribution 4.0 International license.

These experiments were repeated using RNAs incubated with recombinant FLAG-tagged RIG-I protein (Fig. 5C). These results were consistent with the prior experiment: amplification products were present in RIG-IP with RNA from reactivated cells but not from untreated cells or IgG IP (Fig. 5D). To verify the specificity of the RT-PCR, the amplification products were isolated and subjected to Sanger sequencing, and Sanger sequencing results confirmed the specificity of the RT-PCR assay (data not shown).

Since B cells are the primary reservoirs of KSHV and KSHV is tightly associated with primary effusion lymphoma (PEL), we repeated the RIG-I IP and qRT-PCR amplification in biologically relevant, BCBL-1 PEL cells. We used the BCBL1-TRex-RTA cell line since in these cells, similar to iSLK.219 cells, RTA (replication and transcriptional activator) is expressed under the control of a doxycycline-inducible promoter, and viral reactivation can be 100% induced in the majority of cells without simultaneously affecting other cellular signaling pathways and increasing cellular transcription as is the case with phorbol esters and sodium butyrate. The qRT-PCR procedure is illustrated in Fig. 5E. Briefly, BCBL-1-TRex-RTA cells were reactivated with doxycycline and harvested along with mock-treated BCBL-1-TRex-RTA cells. The lysates were incubated with antibodies to endogenous RIG-I or IgG antibodies and subjected to the CLIP procedure. Total RNA was purified from the immunocomplexes and processed for reverse transcription and qPCR (Fig. 5E). The PCR primers used previously for iSLK.219 RIG-I IPs were used in BCBL-1-TRex-RTA cells, since the primer sequences are identical between the iSLK.219 KSHV strain and the KSHV BCBL-1-BAC36 (38) strain, but the nucleotide numbers for each gene are slightly different. Similar to our observation in iSLK.219 cells, PCR established the presence of RNA originating from the KSHV ORF8_10435-10511_, Repeat region (LIR1)_117812-117957_, and ORF25_43265-43354_ regions in RIG-IP samples from reactivated BCBL-1 cells, but not in samples from RIG-IP latent cells or IgG IP samples (Fig. 5F).

In summary, the same viral transcripts were immunoprecipitated with RIG-I from two independent KSHV-positive cell lines: iSLK.219 and BCBL1-TRex-RTA cells. These results are in agreement with the results of the deep sequencing analyses and substantiate the observation that RIG-I interacts with at least three KSHV RNA fragments derived from ORF8_10420-10496_, Repeat region (LIR1)_119059-119204_, and ORF25_43561-43650_ (sequences are shown in Table S2).

10.1128/mBio.00823-18.7 TABLE S2  Sequences of KSHV RNAs based on deep sequencing analysis and qRT-PCR. Download TABLE S2, DOCX file, 0.02 MB. Copyright © 2018 Zhang et al. 2018 Zhang et al. This content is distributed under the terms of the Creative Commons Attribution 4.0 International license.

### Bioinformatic analysis of RIG-I-bound viral RNA fragments.

To determine whether there were any common motifs in the three RNA fragments that most reproducibly copurified with RIG-I [ORF8_10420-10496_, Repeat region (LIR1)_119059-119204_, and ORF25_43561-43650_], we aligned their sequences (shown in Table S2) against each other and could not find any obvious sequence similarity (data not shown). Next, these sequences were subjected to structure prediction using the RNA Mfold program (39). Mfold is quite accurate for small nucleic acid fragments. For instance, it uncovered the structural constraints of microRNAs. The three RNA regions that bound RIG-I were highly structured (Fig. S2), but the data set was too limited to derive any particular motifs. These data are merely suggestive of the notion that RIG-I may detect double-stranded RNA (dsRNA) structures.

10.1128/mBio.00823-18.2 FIG S2  Secondary structural predictions indicate that the identified RNA regions are highly structured. Secondary structure predictions were performed using RNA Mfold program Download FIG S2, TIF file, 1.4 MB. Copyright © 2018 Zhang et al. 2018 Zhang et al. This content is distributed under the terms of the Creative Commons Attribution 4.0 International license.

To characterize the RNA fragments that bound to RIG-I in an unbiased way, we generated a multiple alignment based on all unique sequences in the different samples and then constructed a circular tree to visualize sequence diversity as determined by the Jukes-Cantor metric/neighbor-joining algorithm/100 bootstrap tests (Fig. S3). The sequences obtained from RIG-I IP of doxycycline-treated iSLK.219 cells were much more diverse than the sequences obtained from the IgG IP of the same cells or from a RIG-I IP or IgG IP of mock-treated cells. Note the change in scale of “RIG-I IP:Dox” compared to all others (Fig. S3). This suggests that upon KSHV reactivation, more RNA fragments bound RIG-I and these represent a diverse repertoire in terms of primary sequence. This is consistent with the notion that RIG-I evolved not to bind a specific sequence, but as a sensor for diverse RNA species produced by various virus infections. Finally, the RNA transcripts from reactivated cells bound to RIG-I were more adenosine-and-uridine-rich than the sequences bound to control IgG (Fig. S4). The conditional density plot shows that there was an increased percentage of AU-rich sequences bound to RIG-I in reactivated (Dox) cells compared to control cells even though no particular motif or single gene emerged (Fig. S4). This is consistent with prior studies, which found that RIG-I preferentially binds to AU-rich sequences (19, 40).

10.1128/mBio.00823-18.3 FIG S3  The circular tree shows the diversity of the sequences obtained from RIG-I IP of doxycycline-treated iSLK.219 cells. To visualize the complexity of the RNA population that was immunoprecipitated in each experiment, all small RNA fragment sequences were *de novo* assembled into short contigs to reduce complexity. This yielded the set of unique sequences for each experimental condition. This was followed by multiple alignment (gap open cost of 10; extension cost of 1), and a circular tree of the alignment was constructed for the purpose of visualization. Tree building used the neighbor-joining algorithm with Jukes-Cantor distance measure and 100 bootstrap replicates. Branches are indicated by lines, and terminal leaves are indicated by blue circles. The scale bar indicates relative size for each of the panels. In this representation, a smaller diameter circle and fewer branches indicate a less diverse sequence population. Note that the tree for RIG-I plus Dox (A) is bigger, i.e., representing a more diverse sequence set, not only by visual comparisons with the other conditions (B to D) but also that the scale bars are different. Hence, the set of sequences that bound RIG-I under Dox-induced conditions is very large and diverse. Download FIG S3, TIF file, 0.7 MB. Copyright © 2018 Zhang et al. 2018 Zhang et al. This content is distributed under the terms of the Creative Commons Attribution 4.0 International license.

10.1128/mBio.00823-18.4 FIG S4  RIG-I preferentially binds AU-rich sequences in reactivated cells. Another way to visualize the different sequences bound to RIG-I under Dox-induced conditions is to determine the AU content (as a percentage) for each sequence. The percent AU on the *x* axis for each of the four conditions listed is indicated by four different colors. The *y* axis shows the relative fraction (marginal distributions) for each of the four conditions. Sequences bound to RIG-I under Dox-induced conditions dominate composition at >75% AU, whereas sequences bound to IgG under Dox-induced conditions dominate the composition at <25% AU. Download FIG S4, TIF file, 16.1 MB. Copyright © 2018 Zhang et al. 2018 Zhang et al. This content is distributed under the terms of the Creative Commons Attribution 4.0 International license.

### KSHV ORF8_10420-10496_ RNA induces IFN-β and RIG-I.

**FIG 6:**
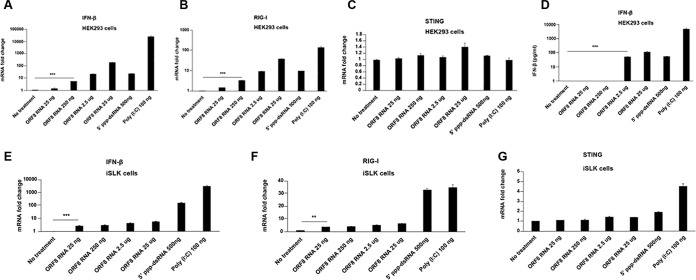
KSHV ORF8_10420-10496_ RNA induces IFN-β and RIG-I in RNA-transfected cells. (A to D) HEK293 cells in 24-well plates were transfected with 5′-ppp-dsRNA or poly(I⋅C) (positive controls), or with the indicated concentration (in nanograms or micrograms) of ORF8_10420-10496_ RNA or not transfected (no-treatment control). At 24 h after transfection, cells were harvested for RNA extraction. The relative mRNA levels of IFN-β (A), RIG-I (B), and STING (C) normalized to β-actin were measured by qRT-PCR. The IFN-β protein level was measured by ELISA from the culture supernatants (D). (E to G) Uninfected iSLK cells in 24-well plates were transfected with 5′-ppp-dsRNA or poly(I·C) (positive controls) or with the indicated concentration of ORF8_10420-10496_ RNA or not transfected (negative control). At 24 h after transfection, cells were harvested for RNA extraction. The relative mRNA levels of IFN-β (E), RIG-I (F), and STING (G) normalized to β-actin were measured by qRT-PCR. Data are presented as mean plus SD. Error bars represent the variation range of duplicate experiments. The data are representative of three independent experiments. **, *P* < 0.01; ***, *P* < 0.001.

Since RNA fragments from multiple regions in the KSHV genome bound RIG-I, we examined the ability of a chemically synthesized ORF8_10420-10496_ RNA to activate RIG-I. Different amounts of ORF8_10420-10496_ RNA were transfected into HEK293 cells. Poly(I·C) and 5′ triphosphate double-stranded RNA (5′-ppp-dsRNA), a RIG-I synthetic ligand, were transfected into HEK293 cells as controls. As seen in Fig. 6A, ORF8_10420-10496_ RNA induced IFN-β transcription in a dose-dependent manner. Interestingly, ORF8_10420-10496_ RNA also induced high levels of RIG-I mRNA in a dose-dependent manner (Fig. 6B) but did not induce any STING (stimulator of interferon genes) mRNA (Fig. 6C). IFN-β mRNA induction detected by qRT-PCR was confirmed by an enzyme-linked immunosorbent assay (ELISA) for secreted IFN-β protein in response to ORF8_10420-10496_ RNA transfection in HEK293 cells (Fig. 6D). To confirm the activity of the ORF8_10420-10496_ RNA fragment in a KSHV-permissive cell line, ORF8_10420-10496_ RNA was transfected into KSHV-negative iSLK cells, and IFN-β mRNA was ascertained at 48 h posttransfection. Similar to the results in HEK293 cells, significantly higher levels of IFN-β (Fig. 6E) and RIG-I (Fig. 6F), but not STING (Fig. 6G), were recorded in iSLK cells.

### IFN-β induction by KSHV ORF8_10420-10496_ RNA is dependent on RIG-I.

**FIG 7:**
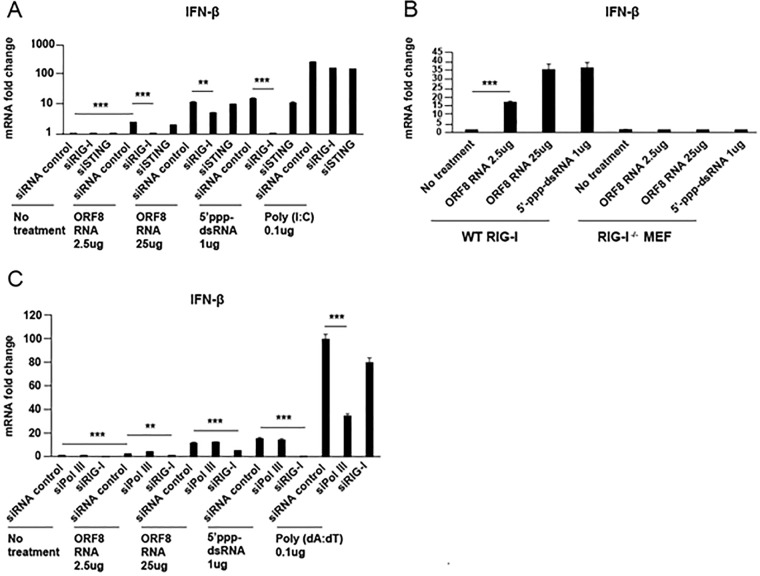
IFN-β induction by KSHV ORF8_10420-10496_ RNA is dependent on RIG-I but not on RNA Pol III. (A) HEK293 cells in 24-well plates were transfected with siRNAs targeting RIG-I or STING. At 48 h posttransfection, cells were transfected with 5′-ppp-dsRNA or poly(I·C) (positive controls), the indicated concentration (in micrograms) of ORF8_10,420-10,496_ RNA, or not transfected (no-treatment control). At 24 h after ORF8_10420-10496_ RNA transfection, IFN-β mRNA was measured by qRT-PCR. (B) WT RIG-I and RIG-I knockout MEF in 24-well plates were transfected with 5′-ppp-dsRNA (a positive control) or with the indicated concentration of ORF8_10420-10496_ RNA or not transfected (no-treatment control). At 24 h after transfection, IFN-β mRNA was measured by qRT-PCR. (C) HEK293 cells in 24-well plates were transfected with siRNAs targeting RNA Pol III or RIG-I. At 48 h posttransfection, cells were transfected with poly(I·C) or poly(dA·dT) (positive controls) or with the indicated concentration of ORF8_10,420-10,496_ RNA or not transfected (no-treatment control). At 24 h after ORF8_10420-10496_ RNA transfection, IFN-β mRNA was measured by qRT-PCR. Data are presented as mean ± SD. Error bars represent the variation range of duplicate experiments. The data are representative of three independent experiments. **, *P* < 0.01; ***, *P* < 0.001.

RNA Pol III was not required for IFN-β induction following primary KSHV infection and/or viral reactivation (Fig. 1). To test the hypothesis that ORF8_10420-10496_ RNA was able to induce IFN-β independent of RNA Pol III or STING, but dependent on RIG-I, *in vitro*-synthesized ORF8_10420-10496_ RNA was transfected into HEK293 cells after transient knockdown of RIG-I, STING, or Pol III. Poly(I·C) and 5′-ppp-dsRNA transfection served as positive controls. As seen in Fig. 7A, compared to nontargeting control siRNA, IFN-β induction was inhibited by RIG-I siRNA depletion but not by STING siRNA depletion, indicating that IFN-β induction by ORF8_10420-10496_ RNA was dependent on RIG-I. Next, we tested IFN-β induction in WT RIG-I and RIG-I^−/−^ MEF that were transfected with different amounts of ORF8_10420-10496_ RNA or 5′-ppp-dsRNA as a positive control. As seen in Fig. 7B, IFN-β was induced by ORF8_10420-10496_ RNA in WT RIG-I cells but not in RIG-I^−/−^ cells. Finally, RNA Pol III and RIG-I were knocked down in HEK293 cells by siRNA transfection followed by transfection of different amounts of ORF8_10420-10496_ RNA. Poly(dA·dT) and 5′-ppp-dsRNA transfection served as positive controls. IFN-β induction was similar in Pol III siRNA- and nontargeting control siRNA-treated cells, but reduced in cells transfected with RIG-I siRNA prior to ORF8_10420-10496_ RNA transfection (Fig. 7C). These results indicate that KSHV ORF8_10420-10496_ induces RIG-I-dependent IFN-β signaling.

### KSHV ORF8_10420-10496_ RNA triggers both NF-κB and IRF3 pathways.

**FIG 8:**
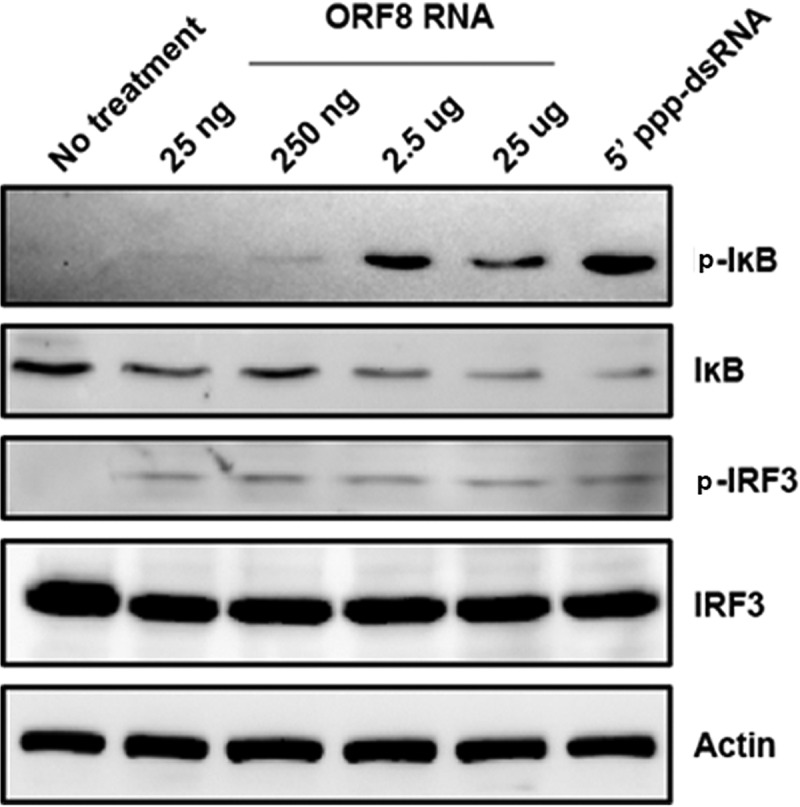
KSHV ORF8_10420-10496_ RNA activates both NF-κB and IRF3. HEK293 cells in 24-well plates were transfected with 5′-ppp-dsRNA (a positive control) or with the indicated concentration of ORF8_10420-10496_ RNA or not transfected (no-treatment control). At 24 h after transfection, cellular protein lysates were analyzed by Western blotting for phosphorylated IκB (p-IκB) and IRF-3 (p-IRF3), total IκB and IRF-3, and actin. The data are representative of two independent experiments.

To test the hypothesis that ORF8_10420-10496_ induced a prototypical RIG-I response, we investigated the activation of transcription factors known to be involved in the induction of IFN-β upon RIG-I activation (41). Specifically, we examined IRF3 phosphorylation and phosphorylation of the inhibitor of κB (IκB). As seen in Fig. 8, IκB phosphorylation occurred in cells transfected with at least 250 ng of ORF8_10420-10496_ RNA. Maximal IκB degradation occurred in cells transfected with 2.5 µg or 25 µg of ORF8_10420-10496_ RNA as well as 5′-ppp-dsRNA. IRF3 was phosphorylated following transfection of 2.5 µg or 25 µg of ORF8_10420-10496_ RNA (Fig. 8). These data suggest that ORF8_10420-10496_ RNA triggers a typical RIG-I response.

## DISCUSSION

Many, structurally diverse RIG-I ligands, such as 5′-ppp-ssRNA (ssRNA stands for single-stranded RNA), short dsRNA, full-length genomes, and poly(U/UC) motifs in the genomes of RNA viruses (HCV, measles, rabies, WNV, and Ebola viruses) activate RIG-I (16, 40, 42, 43). These ligands include folded RNAs of <300 bp (9). Recently, a cellular long noncoding RNA (lncRNA), lnc-Lsm3b, was shown to compete with viral RNAs for RIG-I binding (44). Ablasser et al. (21) previously showed that RIG-I is activated by short nuclear RNAs of EBV named EBERs. KSHV is also a human gammaherpesvirus, and recent whole-genome transcriptome analysis has revealed many diverse RNAs that are transcribed during lytic replication, reactivation from latency, and presumably shortly after primary infection (45). Both lncRNAs and microRNAs are encoded by KSHV (46–49), although a Pol III-dependent analogue of the EBV EBERs has not been reported. One could speculate that these RNAs, many of which may represent unintended or abortive lytic transcripts of unknown secondary structure, are recognized by RIG-I, and our data suggest that this is the case.

We have identified multiple regions in the KSHV genome that encode RNA molecules that interact with RIG-I during the course of KSHV reactivation as well as *in vitro*. We validated three RNA fragments of KSHV [ORF8_10420-10496_, Repeat region (LIR1)_119059-119204_, and ORF25_43561-43650_] that bind to RIG-I. We further showed that synthesized ORF8_10420-10496_ was sufficient to induce RIG-I-dependent IFN-β signaling. These RNA fragments represent a minimal set of potential RIG-I activators and should not be taken as evidence that these are the only regions in the KSHV genome that give rise to RIG-I binding RNAs or that these represent RNA structures with the highest affinity for RIG-I. As far as RIG-I/RNA interactions are concerned, the amount of potential viral versus cellular RNAs bound to RIG-I may be a determining factor for RIG-I activation. This was borne out for KSHV, as the total number of KSHV RNAs associated with RIG-I increased dramatically during viral reactivation.

Alignment results showed that these three fragments do not share any significant sequence similarity, suggesting that RIG-I senses either RNA structures within the KSHV genome or an as yet unidentified stimulatory motif. The KSHV RNA fragments can internally base pair and form RNA secondary structures. Indeed, secondary structural predictions with the RNA Mfold program indicate that the identified stimulatory regions within the KSHV genome are highly structured (see Fig. S2 in the supplemental material). These findings are consistent with the notion that double-stranded structures within viral RNAs function as primary activators of RIG-I during infection (14, 50–52). One intriguing question is whether these structures are also present in other herpesviruses. We cannot exclude the possibility that similar RNA structures exist in the transcripts of other herpesviruses and that these also bind RIG-I and induce type I interferons. As to cellular RNAs, we found that a synthesized GusB RNA fragment, the same length as ORF8_10420-10496_, did not induce any IFN-β when transfected into HEK293 or iSLK cells (data not shown).

We focused on the specific, testable hypothesis that KSHV RNAs bound to RIG-I. Beyond viral transcripts, our analysis also identified cellular RNAs of multiple biotypes that were enriched with either RIG-I or control IgG under conditions of KSHV reactivation. Future studies are needed to understand the mechanism of cellular RNAs bound to RIG-I in the context of KSHV reactivation. It should be noted that in the case of viral infections, where pathogen-derived RNAs take over the total RNA pool during viral replication, there is enough overrepresentation of viral sequences to make individual genome locus assignments on the basis of next-generation sequencing (NGS). The differential is much less pronounced for cellular RNAs.

We also investigated the role of RNA Pol III in the KSHV-mediated induction of the RIG-I pathway. Knockdown or inhibition of RNA Pol III had no effect on IFN-β induction by KSHV infection (Fig. 1), suggesting that different herpesviruses may be detected by RIG-I through different mechanisms.

The three regions of KSHV that we identified as RIG-I binding [ORF8_10420-10496_, Repeat region (LIR1)_119059-119204_, and ORF25_43561-43650_] were present only in the doxycycline-induced reactivated iSLK.219 cells but not in latent iSLK.219 cells (Fig. 4 and 5). This is consistent with our previous publication showing that KSHV-derived dsRNA species are detected in the context of reactivation, but not in latent iSLK.219 cells (33). Using synthetic ORF8_10420-10496_ RNA transfection in cells, we further verified that ORF8_10420-10496_ can trigger the RIG-I-dependent NF-κB and IRF3 signaling pathways (Fig. 6, 7, and 8). A recent study reported that when synthesized stem-loop RNA (SLR) molecules are delivered into mice, they bind more specifically to RIG-I than do poly(I·C) or Sendai virus RNA, which bind to many types of receptors (such as MDA5, TLR3, and TLR7) (53). Our finding provides evidence that viral panhandle RNAs such as ORF8_10420-10496_ may be used to activate RIG-I and induce type I interferon in mice.

In summary, we have determined a mechanism by which RIG-I senses KSHV infection and defined multiple KSHV RNA fragments that bind to RIG-I and induce type I interferon.

## MATERIALS AND METHODS

### Cell culture.

HEK293 cells were maintained in Dulbecco’s modified Eagle medium (DMEM) (Cellgro) containing 10% fetal bovine serum (FBS) and 1% penicillin-streptomycin (Pen-Str). iSLK.219 cells harboring latent rKSHV.219 (r stands for recombinant) were maintained and reactivated as previously described (36). BCBL1-TRex-RTA cells were maintained in RPMI 1640 medium supplemented with 10% tetracycline (Tet)-free FBS, 1% Pen-Str, 1% l-glutamine, 0.075% sodium bicarbonate, and 0.05 mM β-mercaptoethanol. Doxycycline (1 µg/ml) was added to the BCBL1-TRex-RTA cell medium and incubated for 24 h for reactivation. Wild-type (WT) RIG-I and RIG-I^−/−^ mouse embryo fibroblast (MEF) cell lines were kindly provided by Mark Heise (Department of Genetics, University of North Carolina [UNC]). Cells were maintained at 37°C in 5% carbon dioxide.

### RNA transfection and virus infection.

High-molecular-weight polyinosinic-polycytidylic acid [poly(I·C)] (1 mg/ml) and the RIG-I synthetic ligand 5′-triphosphate double-stranded RNA (5′-ppp-dsRNA) (100 µg/ml) were purchased from InvivoGen. Poly(dAT-dTA), and poly(I·C) were transfected at a final concentration of 4 µg/ml, and 5′-ppp-dsRNA was transfected at a final concentration of 1 µg/ml. Kaposi’s sarcoma-associated herpesvirus (KSHV) ORF8_10420-10496_ RNA (5′-UCCUUAUACACCAGAGUCUCGUUGCGGGUGAUGAAGUAGUGUUCGCAGGUGUCUUUGCAGGUUUCCACCUGGUUGUUGGU-3′) was synthesized and purified by Eurofins Genomics (Louisville, KY, USA). RNAs were delivered into the cells with Lipofectamine RNAiMAX (Invitrogen, Carlsbad, CA) according to the manufacturer’s instructions. Vesicular stomatitis virus (VSV) was provided by Douglas Lyles (Wake Forest University School of Medicine, USA). For virus infection, cells were washed with phosphate-buffered saline (PBS) and treated with the culture medium (“mock treated”) or infected with VSV in serum-free and antibiotic-free medium. KSHV was prepared and titrated as previously described (54). The protocol for KSHV infection in HEK293 cells was described previously (55).

### Cross-linking and RIG-I immunoprecipitation (IP).

The iSLK.219 cells were reactivated with doxycycline (Dox), and the unreactivated control iSLK.219 cells were washed with PBS (10 mM phosphate, 137 mM NaCl, 2.7 mM KCl [pH 7.5]) and exposed at 250 ml/cm^2^ to 365-nm UV light using an XL-1000 UV cross-linker (Spectronics Corporation, Westbury, NY). Cells were harvested and incubated in NP-40 lysis buffer (50 mM Tris-HCl [pH 7.5], 150 mM NaCl, 50 mM NaF, 10 mM ZnCl_2_, 0.1% NP-40, 0.5 mM dithiothreitol [DTT], complete protease inhibitor cocktail [Roche]), and 100 U/ml RNaseOUT (Invitrogen) for 15 min on ice. The lysate was cleared by centrifugation to remove cell debris. The resulting supernatants containing endogenous RIG-I were divided equally and incubated with anti-RIG-I (D14G6) rabbit monoclonal antibody (MAb) (catalog no. 3743; Cell Signaling) or anti-IgG (DA1E) rabbit MAb (catalog no. 3900; Cell Signaling) overnight at 4°C on a rotating shaker before adding protein G magnetic beads (Cell Signaling). Two hours later, beads were separated using a magnetic separation rack and were washed based on published techniques for immunoprecipitation (IP) with KSHV specifically (56) and as follows: thrice with cold high-stringency buffer (15 mM Tris-HCl [pH 7.5], 5 mM EDTA, 2.5 mM EGTA, 1% Triton X-100 [TX-100], 1% Na deoxycholate, 0.1% SDS, 120 mM NaCl, 25 mM KCl) and then twice with high-salt buffer (15 mM Tris-HCl [pH 7.5], 5 mM EDTA, 2.5 mM EGTA, 1% TX-100, 1% Na deoxycholate, 0.1% SDS, 1 M NaCl). Bead samples were divided for RNA extraction or subjected to immunoblotting. The purified RNA from each sample was tested for interferon (IFN) stimulatory activity or subjected to Illumina deep sequencing.

For RIG-I purification, FLAG-tagged RIG-I (FLAG-RIG-I) was cloned into pE-SUMOSTAR (Life Sensors), and the plasmid DNA was transformed into E. coli BL21(DE3) RIPL (Agilent). Conditions for expression of RIG-I were taken from a previous published protocol (57). FLAG-RIG-I was purified using HisTrap HP column chromatography (GE Healthcare), followed by HiTrap heparin HP column chromatography (GE Healthcare). Superdex 200 chromatography (GE Healthcare), anti-FLAG M2 magnetic beads (Sigma), and a final Superdex 200 chromatographic step were used to further purify FLAG-RIG-I.

For IP using purified RIG-I, 10 µg of purified FLAG-tagged RIG-I protein was incubated with RNAs extracted from reactivated or unreactivated iSLK.219 cells in ice-cold buffer C (0.5% NP-40, 20 mM Tris-HCl [pH 7.5], 150 mM NaCl, 2.5 mM MgCl_2_), complete protease inhibitor cocktail (Roche), and 100 U/ml RNaseOUT (Invitrogen) for 2 h at 4°C on a rotating shaker. The reaction mixtures were further incubated with anti-FLAG M2 antibody (Sigma-Aldrich) or control mouse IgG1 (mIgG1) (Sigma-Aldrich) overnight at 4°C on a rotating shaker before adding Gamma Bind Plus Sepharose beads (GE Healthcare). The rest of the IP was performed the same way as the endogenous RIG-I IP.

### RNA extraction, qRT-PCR analysis, and ELISA.

The immunoprecipitated RNAs were extracted using TRIzol (Invitrogen) according to the manufacturer’s protocol. Total RNA from RNA-transfected or virus-infected RIG-I MEF was isolated according to the manufacturer’s protocol (RNeasy plus kit; Qiagen). The same amount of RNA from each sample was processed for reverse transcription and quantitative PCR using SuperScript III reverse transcriptase (Invitrogen), SYBR green PCR master mix (Bio-Rad), and ABI 7300, as described previously (58). Primer sequences for beta interferon (IFN-β), RIG-I, and actin were described previously (33). Sequences and additional primers targeting different KSHV regions, including ORF8, Repeat region (LIR1), and ORF25, were described in Tables S1 and S2 in the supplemental material. Culture supernatants were collected and subjected to ELISA with a human IFN-β kit (PBL Assay Science, Piscataway, NJ) according to the manufacturer’s instructions.

### Deep sequencing of RNA.

Next-generation sequencing and library preparation were performed on the Illumina HiSeq2500 system (rapid-run mode) in the High Throughput Sequencing Facility (HTSF) at UNC-Chapel Hill. RNA was purified and treated with RNase-free DNase to remove any genomic DNA contamination (Zymo Research). The RNA concentration was estimated using Qubit methodology (Invitrogen). Then, the RNA was prepared for Illumina sequencing using a modified small RNA sequencing kit (NEXTflex small RNA sequencing kit V3) according to the manufacturer’s protocol (Perkin-Elmer). The ligated cDNA was amplified using 24 PCR cycles. Prepared libraries were pooled for DNA sequencing. DNA concentration of prepared libraries and pool was performed using a broad-range Qubit (Invitrogen) kit, and a profile of DNA size was established by Bioanalyzer QC DNA 1000 chip (Agilent). A single-end 100-cycle sequencing run was used for the analyzed pool on HiSeq2500 rapid mode. The obtained sequences were processed with the FASTX toolkit (http://hannonlab.cshl.edu/fastx_toolkit/) in order to remove adaptor sequences and reads with PHRED scores below 30. Alignment to the KSHV strain JSC-1 BAC16 genome (GenBank accession no. GQ994935.1) or the BCBL-1-BAC36 genome (GenBank accession no. HQ404500) (38) was performed using CLC bio (Qiagen Inc.). Relative sequence abundances were analyzed for RIG-I pulldown samples and the IgG control. Specific read enrichments were calculated by determining the relative sequence abundance at each position on the genomic segment and calculating the average of the RIG-I/IgG ratios. Further processing was done using custom R code available at https://bitbucket.org/ddittmer/rig-i-ip/src/master/. The raw reads are available from SRA archives under BioProject accession no. PRJNA451459 and SRA accession no. SRP145129.

### RNA secondary structure prediction.

The RNA Mfold program (39) was used for the prediction of secondary structures.

### RNA interference and immunoblotting.

The small interfering RNA (siRNA) negative control (D-001810-10-05), siRNAs targeting human RIG-I (Ddx58) (L-012511-00-0005), and the two subunits of RNA polymerase (Pol) III (Polr3A and Polr3D) (L-019741-01-0005 and L-011594-00-005) were purchased from Dharmacon. For knockdown of target genes, siRNAs were transfected into the cells with Lipofectamine RNAiMAX (Invitrogen) according to the manufacturer’s recommendations. At 48 h or 72 h posttransfection, cells were harvested or infected with viruses for further experiments. For immunoblotting, cells were washed with PBS and lysed in 0.1% NP-40 buffer plus protease inhibitor complete tablets (Roche). Equal amounts of lysate were separated using SDS-PAGE and transferred onto Hybond ECL nitrocellulose membranes (GE Healthcare). The antibodies used included anti-RIG-I (D14GG) (Cell Signaling), anti-RNA Pol III (POLR3D) (Sigma), anti-IRF3 (D83B9) (Cell Signaling), anti-phospho-IRF3 (S396) (Cell Signaling), anti-IκB-alpha (H-4) (sc-1643; Santa Cruz), anti-phospho-IκB-alpha (Ser32) (14D4) (2859; Cell Signaling), horseradish peroxidase (HRP)-labeled antitubulin (Cell Signaling), and antiactin (Santa Cruz).

### Statistical analysis.

An unpaired two-tailed Student’s *t* test was used to determine statistically significant differences. *P* values of less than 0.01 or 0.001 were considered statistically significant.

10.1128/mBio.00823-18.5 FIG S5  Summary of the mapping results of two additional independent repeats of Fig. 4. The RIG-I and IgG immunoprecipitations and controls were repeated multiple times, and this figure shows two additional repeats. RNA eluted from the RIG-I or IgG immunoprecipitates was used to generate 100-bp, single-end Illumina libraries. Each library was split into two lanes of an HS2500 to guard against lane effects. All reads were adaptor depleted and error corrected using BBmap, and human sequences were depleted. The remainder sequences were aligned to the KSHV genome using CLC v.11.0.1, and expression values (in reads per kilobase per million [RPKM]) were recorded for each annotated open reading frame (ORF), allowing a maximum number of 10 simultaneous hits per sequence. The color scheme uses red for high RPKM per ORF, white for low RPKM, and blue for no hits. Genome coordinates are indicated at the top of the figure, and ORFs are indicated by arrows. Download FIG S5, TIF file, 6.7 MB. Copyright © 2018 Zhang et al. 2018 Zhang et al. This content is distributed under the terms of the Creative Commons Attribution 4.0 International license.
