# Supplementary figures and images for: RIG-I Detects Kaposi’s Sarcoma-Associated Herpesvirus Transcripts in a RNA Polymerase III-Independent Manner
Source: mBio. 2018 Jul 3;9(4):e00823-18. doi: 10.1128/mBio.00823-18 (PMC6030556; doi:10.1128/mBio.00823-18)

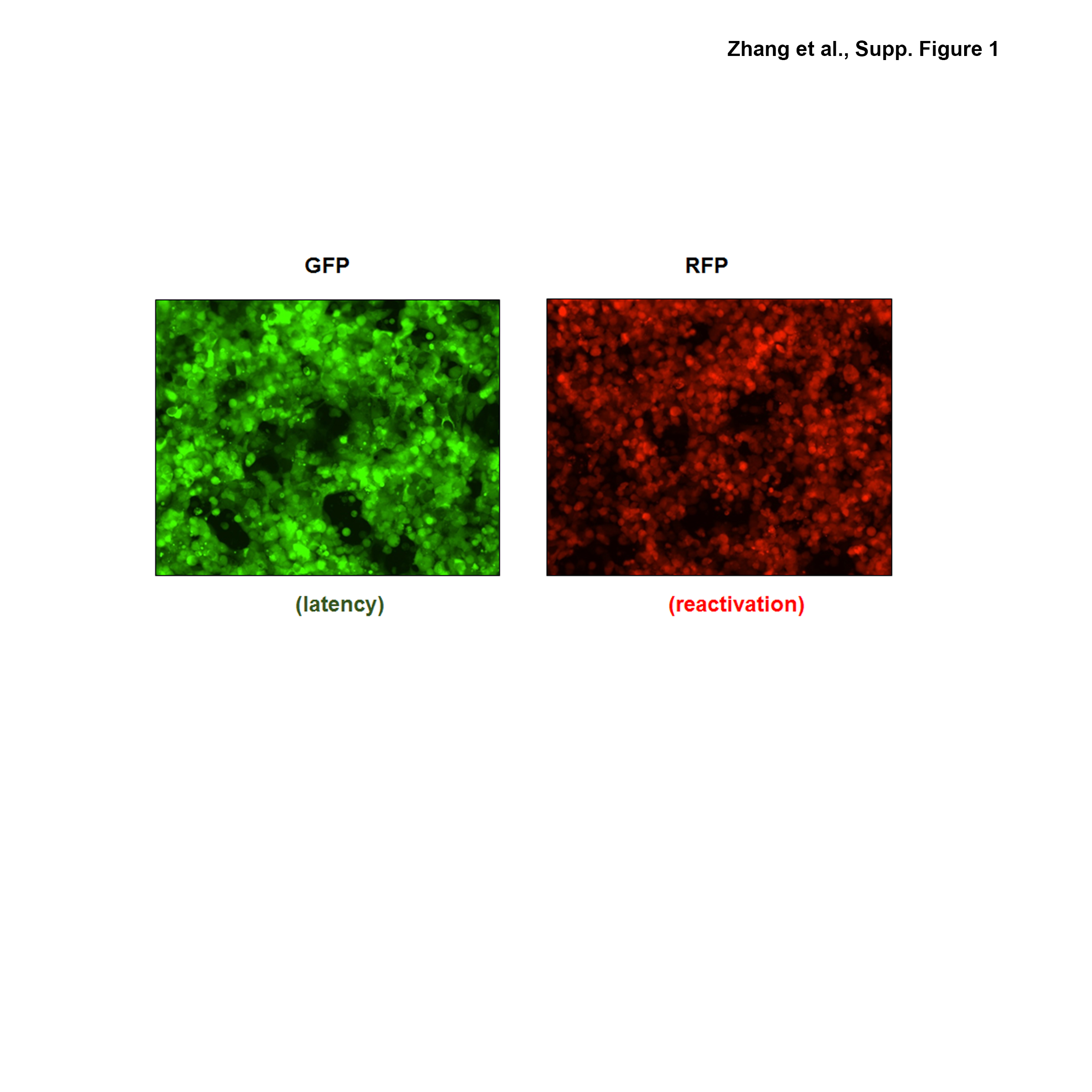

Supplement: FIG S1 [file mbo003183954sf1.tif]

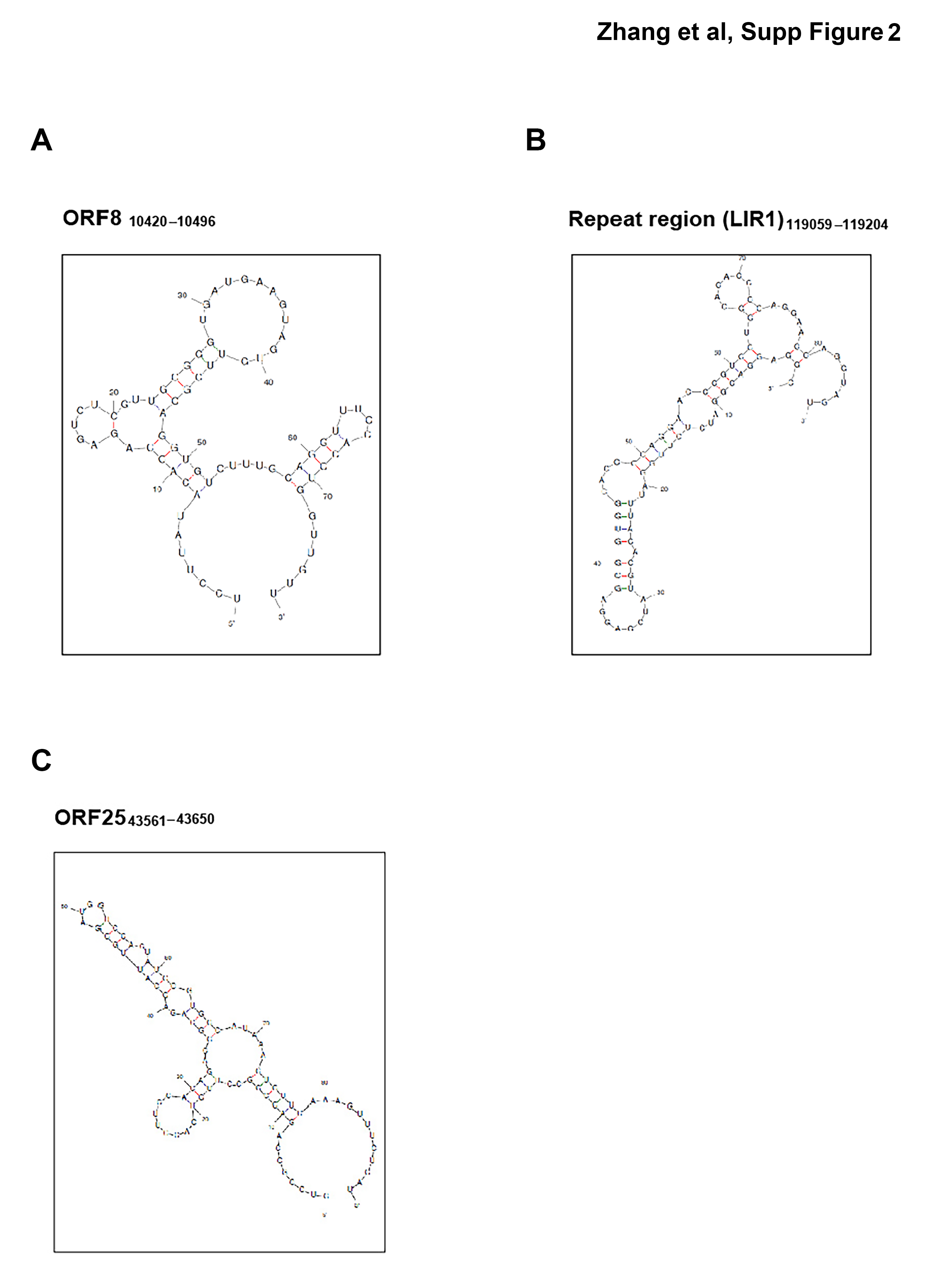

Supplement: FIG S2 [file mbo003183954sf2.tif]

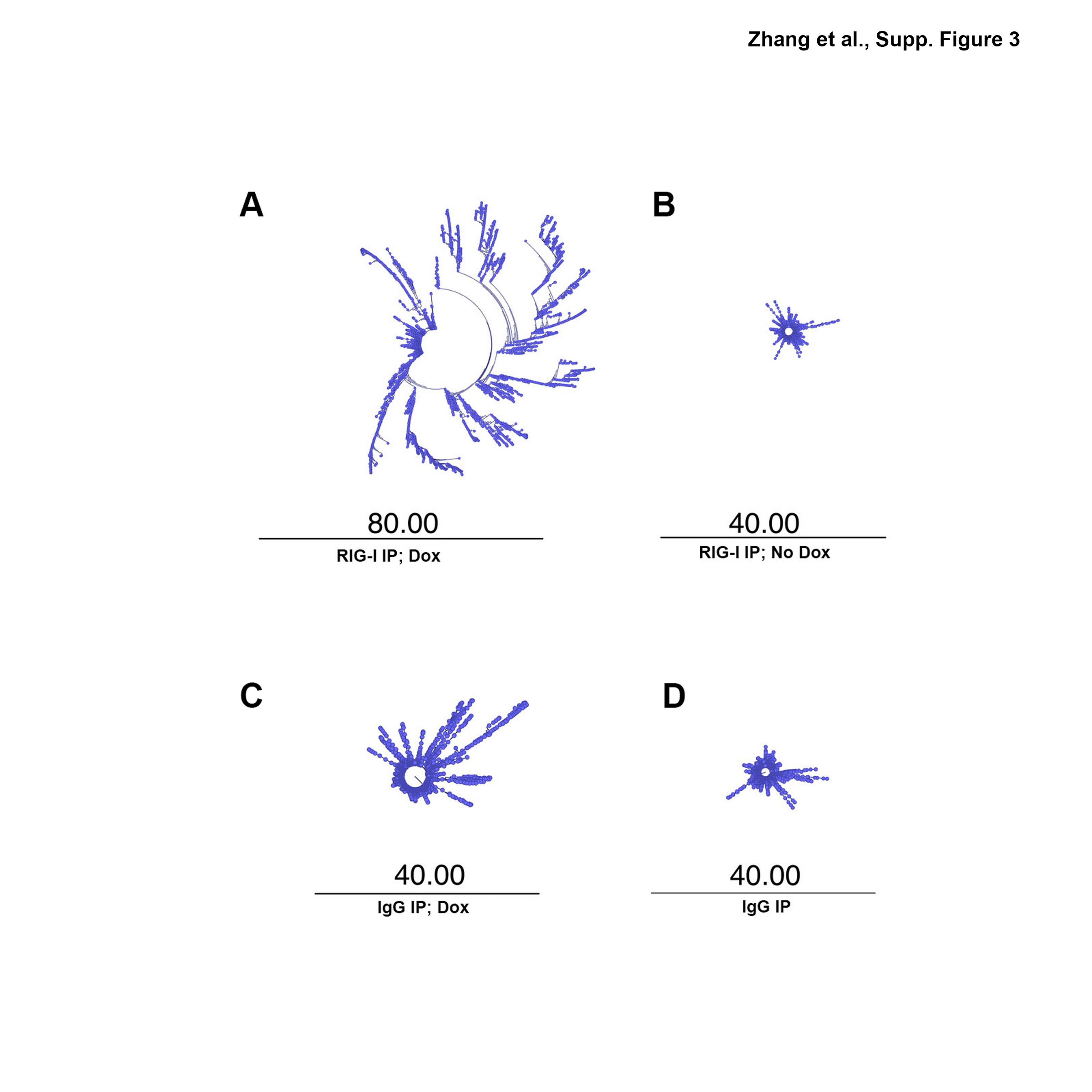

Supplement: FIG S3 [file mbo003183954sf3.tif]

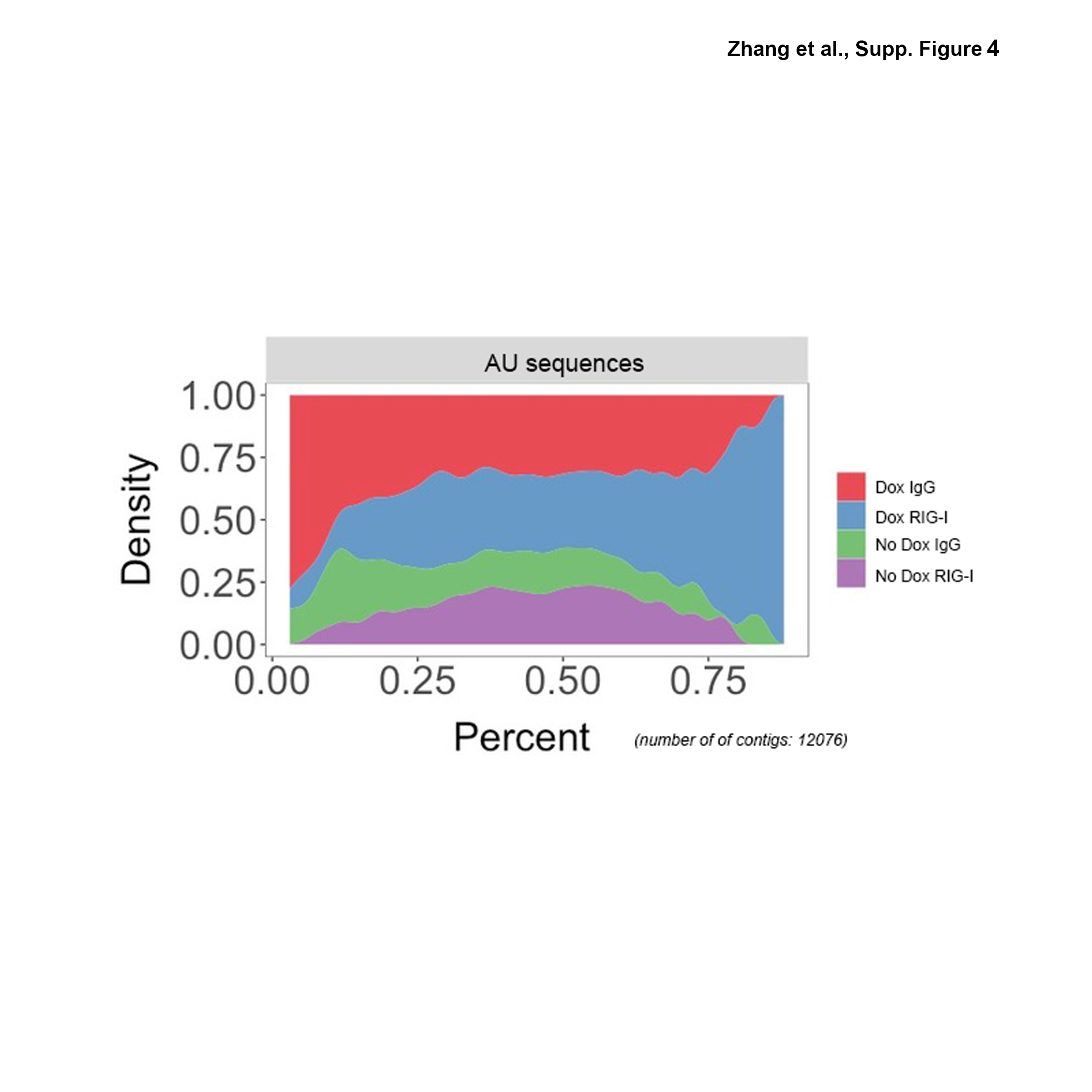

Supplement: FIG S4 [file mbo003183954sf4.tif]
