# Supplementary material for: RIG-I Detects Kaposi’s Sarcoma-Associated Herpesvirus Transcripts in a RNA Polymerase III-Independent Manner
Source: mBio. 2018 Jul 3;9(4):e00823-18. doi: 10.1128/mBio.00823-18 (PMC6030556; doi:10.1128/mBio.00823-18)
Supplement: TABLE S1 [file mbo003183954st1.docx]

**Supplemental Table 1: DNA oligo primers used in qRT-PCR**

| **Name of**  **Genes** | **Forward primers (5’-3’)^1^** | **Reverse primer (5’-3’)** |
| --- | --- | --- |
| ORF8 | ACCAACAACCAGGTGGAAAC | GTCTCGTTGCGGGTGATG |
| Repeat region (LIR1) | GGGTTCCTGGGGTGCCAC | ACGGAGGACGGATCTCTTG |
| ORF25 | TGAACAGTTTATGGCACGCATAGT | gtaACAGACGGCAGAGCATTGG |

^1^Sequences are based on GQ994935

^2^The 5’ first 3 nucleotides (lowercase) are present in some KSHV isolates but not in GQ994935. The underlined region is directly repeated at 63993- 63982.
