# Supplementary material for: RIG-I Detects Kaposi’s Sarcoma-Associated Herpesvirus Transcripts in a RNA Polymerase III-Independent Manner
Source: mBio. 2018 Jul 3;9(4):e00823-18. doi: 10.1128/mBio.00823-18 (PMC6030556; doi:10.1128/mBio.00823-18)
Supplement: TABLE S2 [file mbo003183954st2.docx]

**Supplemental Table 2: Sequences of KSHV RNAs based on deep sequencing analysis and qRT-PCR.**

The gene annotation with the exact nucleotide position on the KSHV genome GQ994935 is shown in brackets.

| **Names of KSHV RNAs** | **Sequences of KSHV RNAs** |
| --- | --- |
| ORF8_10, 420-10,496_  (10,420-10,496) | 5’-UCCUUAUACACCAGAGUCUCGUUGCGGGU  GAUGAAGUAGUGUUCGCAGGUGUCUUUGCAGGUUUCCACCUGGUUGUU-3’ |
| Repeat region (LIR1)_119059-119204_ (119,059-119,204) | 5’-CGGAGGACGGAUCUCUUGGAUUUACACGUA  UCGAGGAGCGGUGGCACCCCAGGAACCCGUCCUGGCACACCCCAGGAACCAGGUAGU-3’ |
| ORF25 _43561-43650_  (43561-43650) | 5’-GUCCGCCAGAGCGGCCUUGUCACCUUCCAC  AGACGGCAGAGCAUUGGGAUGGUCCACUAUGCGUGCCAUAAACUGUUCAAAGUUUCUCAU-3’ |

**Supplemental Table 1: DNA oligo primers used in qRT-PCR**

119124 – 119204

10420 – 10496

43561 – 43650

110584 – 110675

2435 – 2530

16112 – 16159

31205 – 31255

37181 – 37261

53549 – 53613

51896 – 51959

68836 – 68889

133617 – 133712

17792 – 17834

25711 – 25793

74774 – 74965
